# Improving the normalization of complex interventions: part 1 - development of the NoMAD instrument for assessing implementation work based on normalization process theory (NPT)

**DOI:** 10.1186/s12874-018-0590-y

**Published:** 2018-11-15

**Authors:** Tim Rapley, Melissa Girling, Frances S. Mair, Elizabeth Murray, Shaun Treweek, Elaine McColl, Ian Nicholas Steen, Carl R. May, Tracy L. Finch

**Affiliations:** 1 Department of Social Work, Education and Community Wellbeing, Northumbria University, Coach Lane Campus West, Newcastle upon Tyne, NE7 7XA UK; 2 Institute of Health & Society, Newcastle University, Baddiley-Clark Building, Richardson Road, Newcastle-upon-Tyne, NE2 4AX UK; 3 Institute of Health and Wellbeing, University of Glasgow, 1 Horselethill Road, Glasgow, G12 9LX UK; 4 Research Department of Primary Care and Population Health, University College London, Upper Floor 3, Royal Free Hospital, Rowland Hill Street, London, NW3 2PF UK; 5 Health Services Research Unit, University of Aberdeen, 3rd Floor, Health Sciences Building, Foresterhill, Aberdeen, AB25 2ZD UK; 6 Faculty of Public Health and Policy, London School of Hygiene and Tropical Medicine, 15-17 Tavistock Place, London, WC1H 9SH UK; 7 Department of Nursing, Midwifery and Health, Northumbria University, Coach Lane Campus West, Newcastle upon Tyne, NE7 7XA UK

**Keywords:** Normalization process theory, NPT, Implementation process, Questionnaire, Instrument development, Complex interventions, NoMAD

## Abstract

**Background:**

Understanding and measuring implementation processes is a key challenge for implementation researchers. This study draws on Normalization Process Theory (NPT) to develop an instrument that can be applied to assess, monitor or measure factors likely to affect normalization from the perspective of implementation participants.

**Methods:**

An iterative process of instrument development was undertaken using the following methods: theoretical elaboration, item generation and item reduction (team workshops); item appraisal (QAS-99); cognitive testing with complex intervention teams; theory re-validation with NPT experts; and pilot testing of instrument.

**Results:**

We initially generated 112 potential questionnaire items; these were then reduced to 47 through team workshops and item appraisal. No concerns about item wording and construction were raised through the item appraisal process. We undertook three rounds of cognitive interviews with professionals (*n* = 30) involved in the development, evaluation, delivery or reception of complex interventions. We identified minor issues around wording of some items; universal issues around how to engage with people at different time points in an intervention; and conceptual issues around the types of people for whom the instrument should be designed. We managed these by adding extra items (*n* = 6) and including a new set of option responses: ‘not relevant at this stage’, ‘not relevant to my role’ and ‘not relevant to this intervention’ and decided to design an instrument explicitly for those people either delivering or receiving an intervention. This version of the instrument had 53 items. Twenty-three people with a good working knowledge of NPT reviewed the items for theoretical drift. Items that displayed a poor alignment with NPT sub-constructs were removed (*n* = 8) and others revised or combined (n = 6). The final instrument, with 43 items, was successfully piloted with five people, with a 100% completion rate of items.

**Conclusion:**

The process of moving through cycles of theoretical translation, item generation, cognitive testing, and theoretical (re)validation was essential for maintaining a balance between the theoretical integrity of the NPT concepts and the ease with which intended respondents could answer the questions. The final instrument could be easily understood and completed, while retaining theoretical validity. NoMAD represents a measure that can be used to understand implementation participants’ experiences. It is intended as a measure that can be used alongside instruments that measure other dimensions of implementation activity, such as implementation fidelity, adoption, and readiness.

## Background

In the healthcare context, understanding of implementation processes is key to ensuring that innovations are both implemented and sustained in practice. For innovations in service delivery and organisation, desired outcomes of a ‘successful’ implementation are likely to include improvements in efficiency, cost savings, and improved health outcomes or experiences of service users [1]. Despite a vast body of literature on the implementation of complex health innovations, the gap between research evidence and efforts at implementing new technologies and practices remains wide [2].

In the field of implementation science, a range of theories and models have been developed to address these ‘problems of translation’ [3]. There are several influential syntheses of this conceptual literature [4–7] as well as a shift towards the *measurement* of implementation processes proposed by such frameworks [8–11].

The application of implementation science approaches to the development and evaluation of complex healthcare interventions is additionally challenging [12]. Given this complexity, the measurement of implementation processes is a key challenge facing implementation researchers [9, 13–16]. Thus far, many attempts to develop instruments to measure change have been limited in both reliability and validity [9] and are not theory-based [17]. More recent work to develop instruments for measuring implementation processes has brought to the forefront the need to develop brief, reliable and valid instruments to enable testing of theories and to consider the wider implications of the individual as the vehicle for change [14, 17, 18].

A key practical requirement for use of measures in diverse implementation settings is that they can adaptable for use in real service contexts. As such, we follow Glasgow and Riley’s [18] conception of ‘pragmatic measures’ of implementation. They argue for measures of implementation activity that are robustly developed (preferably on the basis of appropriate theory) to meet fundamental psychometric expectations, but which balance this against the practical (usability) needs of stakeholders working in real life implementation environments.

This study extends Normalization Process Theory (NPT) [19] to develop an instrument that can be applied to assess, monitor or measure factors likely to affect normalization from the perspective of implementation participants. NPT is now an established [20] middle-range theory of implementation that explains the normalisation of changes in practice with reference to the complex and collaborative work involved in implementation activities. Support from empirical research continues to grow [21–39], although this remains mostly generated through qualitative studies [20]. Quantitative assessments of NPT are still lacking and to our knowledge, an NPT based measurement instrument has not yet been developed.

Some exploratory work on translating NPT constructs into structured instruments has already been conducted [40–42]. In our previous work, we developed a simple 16-statement ‘toolkit’ containing items representing the theoretical constructs of the NPT for use as a ‘sensitizing tool’ by individuals involved in planning and implementing complex interventions to think through which aspects of their interventions might affect their successful normalization [43]. Although developed through intensive item-development and user feedback activities, the tool was neither developed for use as a research instrument nor validated for this purpose. The NoMAD study extends this work to provide a tool representing NPT, but which is validated for the purposes of measuring participants’ experiences of working collaboratively to implement change over time and across settings, both for advancement of research and evaluation and for practical implementation in intervention settings.

This paper describes the development phase of the NoMAD study, which aimed to develop and validate an NPT based instrument for measuring factors likely to affect normalization from the perspective of implementation participants [44]. We wanted to design an instrument that could be used alongside a range of existing outcome, process and impact measures used within implementation work that would focus on specific aspects of the experiences of participants. Here, we aim to (1) describe the development of the NoMAD instrument, and (2) identify implications for advancing theory-based assessment of implementation process in complex health interventions. A companion paper [45] presents the methods and results of the validation phase of the study, and the final NoMAD instrument.

## Methods

**Fig. 1:**
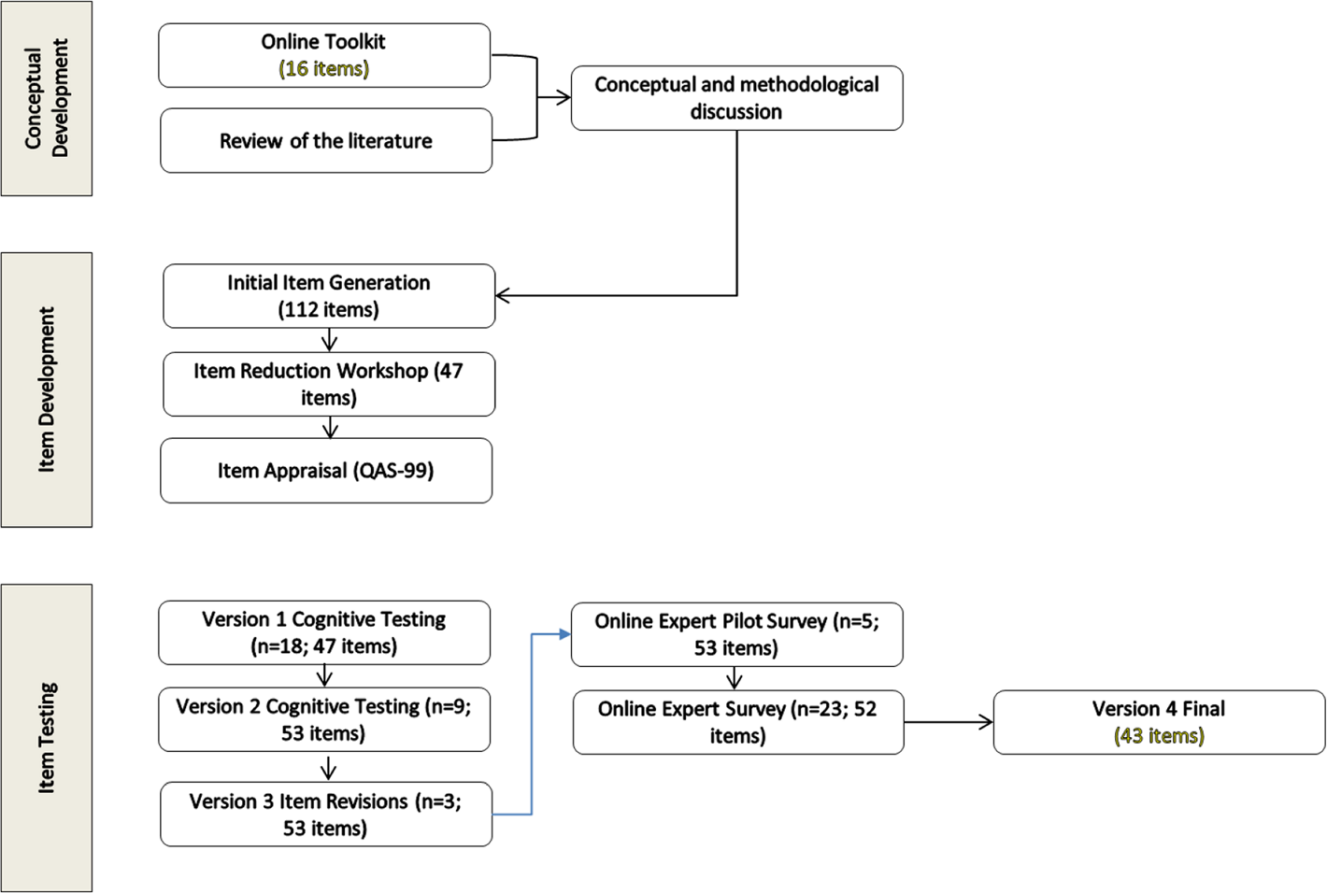
Overview of questionnaire development process

Instrument development methods comprised group work and consensus methods, application of item appraisal tools, cognitive interviews, piloting and expert critique. An overview of the process is presented in Fig. 1. These activities were conducted between March 2012 and February 2014, and involved an iterative process.

### Phase 1: Item development [April 2012 – September 2012]

#### Item generation

As a starting point for item development, we used the 16-item toolkit previously published by the authors [43] (Table 1) alongside returning to the core set of NPT papers which plot its development and potential application [19, 43, 46, 47]. In addition to this, we reviewed the NPT literature to see how authors had operationalized specific constructs and sub-constructs. We then held a face-to-face meeting, over 2 days, with the research team in order to develop a broader set of items to further reflect the theoretical and sub-constructs of the existing toolkit. In this phase, we focused on creating at least three items from each original sub-construct. Our initial aim was to develop items for a questionnaire that could be used in a range of contexts, at a range of time points in the life of an intervention, as well as being completed by a range of people, including researchers involved in developing and evaluating an intervention alongside those people on the ground either delivering or receiving an intervention. As we outline below, during the course of the work, we had to refine our aim, in terms of the target range of people completing the questionnaire.

**Table 1:** Original 16 NPT toolkit items

Coherence		Collective Action	
Sub-construct	Original NPT toolkit item	Sub-construct	Original NPT toolkit item
Differentiation: Whether the [intervention] is easy to describe to participants and whether they can appreciate how it differs or is clearly distinct from current ways of working.	Participants distinguish the intervention from current ways of working	Initiation: Whether or not key individuals are able and willing to get others involved in the new practice.	Key individuals drive the intervention forward
Communal specification: Whether participants have or are able to build a shared understanding of the aims, objectives, and expected outcomes of the proposed [intervention].	Participants collectively agree about the purpose of the intervention	Legitimation: Whether or not participants believe it is right for them to be involved, and that they can make a valid contribution	Participants agree that the intervention is a legitimate part of their work
Individual specification: Whether individual participants have or are able to make sense of the work – specific tasks and responsibilities - the proposed [intervention] would create for them.	Participants individually understand what the intervention requires of them	Enrolment: The capacity and willingness of participants to organize themselves in order to collectively contribute to the work involved in the new practice.	Participants buy in to delivering the intervention
Internalization: Whether participants have or are able to easily grasp the potential value, benefits and importance of the [intervention].	Participants construct potential value of the intervention for them/their work	Activation: The capacity and willingness of participants to collectively define the actions and procedures needed to keep the new practice going.	Participants continue to support the intervention
Cognitive Participation		Reflexive Monitoring	
Sub-construct	Original NPT toolkit item	Sub-construct	Original NPT toolkit item
Interactional Workability: Whether people are able to enact the [intervention] and operationalise its components in practice	Participants perform the tasks required by the intervention	Systematization: Whether participants can determine how effective and useful the [intervention] is from the use of formal and/or informal evaluation methods	Participants access information about the effects of the intervention
Relational Integration: Whether people maintain trust in the [intervention] and in each other.	Participants maintain their trust in each other’s work and expertise through the intervention	Communal appraisal: Whether, as a result of formal monitoring, participants collectively agree about the worth of the effects of the [intervention]	Participants collectively assess the intervention as worthwhile for others
Skill set Workability: Whether the work required by the [intervention] is seen to be parcelled out to participants with the right mix of skills and training to do it	The work of the intervention is appropriately allocated to participants	Individual appraisal: Whether individuals involved with, or affected by, the [intervention], think it is worthwhile.	Participants individually assess the intervention as worthwhile for themselves
Contextual Integration: Whether the [intervention] is supported by management and other stakeholders, policy, money and material resources.	The intervention is adequately supported by its host organization	Reconfiguration: Whether individuals or groups using the [intervention] can make changes as a result of individual and communal appraisal.	Participants modify their work in response to their appraisal of the intervention

#### Item reduction

The initial list of potential items was then distributed to research team members for feedback. We sent each team member two constructs, with the list of potential items for each construct. We then asked them to rank their preferences and to add any additional feedback. So for each construct a minimum of two sets of preferences were obtained. We again met as a team to further discuss which items should be retained and which rejected.

#### Item appraisal

Draft items were systematically tested using the QAS-99 appraisal system [48] and the Question Understanding Aid (QUAID) [49]. The QAS-99 is a question appraisal system that is widely used to evaluate survey questions to ‘find and fix’ questions before going into the field.

### Phase 2: Item testing (September 2012 – July 2013)

Testing item comprehension: Cognitive interviews were undertaken to test the validity and acceptability of the draft items. The cognitive interviewing technique of ‘think aloud’ is widely used in the development of questionnaires [50] to identify and address potential problems of item comprehension and response selection. The ‘think aloud’ technique aims to ‘elicit data on participants’ thought processes as they respond to questionnaire items’ [51]. Three rounds of cognitive interviewing took place between October 2012 and July 2013. The purpose of the interviews was to enable us to review items in turn, and aid decisions as to whether to add new items, revise existing ones, or drop those that could be considered redundant or lacking face validity. Undertaking this process over three rounds meant we could test adjustments and refinements made in light of the findings of prior rounds.

All interviewees were involved in complex health interventions, either in terms of the development, evaluation or delivery thereof. They were identified through various publicly available research databases and via research team contacts. We did not approach anyone who had contributed to the development of NPT in previous work or who had been an author of published studies that had used NPT as a framework, since we wanted to avoid testing the items with people who had a working knowledge of NPT. We felt this was important as for the final questionnaire to be workable, it would have to be comprehensible to those without any prior knowledge of the theory embedded in the questionnaire. Potential participants were initially contacted via email. In some cases, invitees who did not feel that they were suitable to take part referred the email on to colleagues or made suggestions of who to contact. Participants were included from a number and a range of different interventions (Table 2). Six of the participants were Early Career Researchers and/or Practitioners, eighteen mid-career and six senior. They came from diverse clinical backgrounds including General Practice, Health Visiting, Healthcare Assistants, Midwifery, Nursing, Occupational Therapy, Public Health and Speech and Language Therapy and a range of social and behavioural science backgrounds, including Education, Psychology and Sociology.

**Table 2:** Cognitive Interview Participants

	Role		
	Academic	Researcher	Practitioner
Context of Complex Intervention			
Primary Care	2		
Secondary Care		1	3
Public/Community Health	2	7	9
Education	4		2

One team member (MG) conducted face-to-face cognitive interviews with the participants at various locations across the UK. These interviews lasted approximately 20–60 min and were divided into two parts. In the first part, participants were asked to think about a specific complex intervention in which they were previously involved. They were then given a draft version of the questionnaire, containing 47 items. The items were arranged to represent the four NPT constructs in separate consecutive sections, each accompanied by a brief definition of the section construct. Participants were then asked to go through each section, read aloud each item, and think about the intervention they were involved in, specify their level of agreement on a 5-point Likert scale (strongly agree, agree, neither agree nor disagree, disagree, strongly disagree). They were asked to explain their reasoning for giving a particular answer as well as to voice any concerns with their understanding of the items, such as issues around wording or meaning. The second part of the interview involved a number of additional open-ended questions which were designed to elicit information about participants’ engagement in implementation research, to inform wider aspects of the study. These questions related to participants’ expertise and experience of implementing complex interventions; motives for participating in our study; and any application of theories, models and outcome measurement utilised in their own work. Interviews in rounds one and two were recorded and transcribed verbatim. In round three, interviews were recorded but not transcribed. Written consent obtained was obtained in all three rounds.

The cognitive interview transcripts provided data for formal analysis that focused on developing an in-depth understanding of participants’ interpretations of the items. Prescribed guidelines for analysing and interpreting data from cognitive interviews and incorporating cognitive interview feedback in decisions about retention, revision or elimination of potential items is lacking [50]. The core research team (MG, TF & TR) developed an initial coding framework on a subset of data, then extended it as analysis progressed. This coding frame reflected a taxonomy of interpretive approaches to the items. For each participant, transcribed responses were tabulated by question item for independent coding by the three core team members. Independent coding was then discussed in weekly study meetings, and codes (per item, per participant) were agreed and recorded on team-coding sheets. This approach allowed us to explore issues of interpretation both across items (e.g. response tendencies of individuals), and across participants (e.g. identification of patterns of responding to particular items). As the analysis progressed, the initial coding themes were extended and refined as (1) new issues emerged; (2) layers of complexity were uncovered and (3) more in-depth understanding of issues evolved. This final refined coding framework was then re-applied to all of the interview data (see Table 3).

**Table 3:** Cognitive Interviews Coding Framework

Code	Description
‘Got it’	The participant shows good understanding of the question and answers appropriately and confidently, according to their role within the intervention
Wording	The participant queries a particular word or terminology within the question, e.g. unsure of meaning
Redundancy	The participant either suggests that another question in the toolkit is a ‘better’ question to ask; or that they ‘like’ a particular question over another (NB this is only relevant to duplicate questions that have similar wording)
Not Enough Information	The participant does not offer enough information to make a judgement on, for example, understanding or relevance of the question
Problem Of Relevance - ROLE	The participant does not consider the question ‘relevant’ to their role in the intervention e.g. something that is not applicable to them
Problem of relevance - TIMING	The participant does not consider the question ‘relevant’ to the timing of the intervention e.g. hasn’t happened yet
Who	The participant has some trouble with ‘who’ the question is relating to e.g. themself, or others (and who the ‘others’ may be)
Multiple Interpretations - within	The participant offers a response from their own perspective/experience/role as well as that of others involved in the intervention
Multiple Interpretations - across	The participant offers a response which interprets the question as something different to what is being asked

To test the reliability and consistency of the coding framework, data subsets were distributed to five other research team members for review. This further layer of data coding and any discrepancies were incorporated into decisions about final response coding. A series of face-to-face workshops and teleconferences with the study team included detailed discussions of the coded data and subsequent refinement of the items for inclusion in subsequent versions of the instrument during the development process.

#### Testing theoretical integrity

A key objective of the project was to retain theoretical integrity of each of the items with respect to NPT. To check for, and address, possible ‘theoretical drift’ of the items generated from the original NPT constructs in the development stages described above, an online survey, similar to that utilised in previous work [40] of researchers using NPT, was undertaken.

Participants in this on-line critique were selected according to the sampling criterion of either (a) contributing to the development of NPT in previous work and/or (b) being key authors of published studies that had used NPT as a framework. Participants (*n* = 30) were invited to take part in the survey via email, with the questionnaire being delivered using the online survey tool Qualtrix™. Individuals were asked to provide feedback about the relationship between items and the theoretical concepts each intended to represent via a series of rank-order questions.

Firstly, respondents were asked to rate items against the specific theoretical construct (e.g. coherence) that the item was meant to express. We were in interested in how strongly participants felt each item reflected the core construct. We used a standard 5 point Likert-scale, with the anchors from ‘very weak’ to ‘very strong’, with the mid-point being ‘moderate’. As respondents had a working knowledge of NPT, we did not include a ‘don’t know’ option. To limit order effects, all the respondents received a randomised version of the items within each construct. Secondly, respondents were asked to align items with the theoretical sub-constructs (see Table 4 for an example). They had to select the sub-construct that they felt was best represented by the item. We also offered them the choice to choose more than one sub-construct as well as choosing no sub-construct, as we were keen to explore if any items where either too broad or felt to be in some way inappropriate.

**Table 4:** Alignment of items with theoretical sub-constructs: Example of Coherence item

	Response				
Example Question	Sub-Construct A E.g. Differentiation: Whether people can easily understand that the intervention is different from current ways of working	Sub-construct B E.g. Communal specification: Whether people can build a collective understanding of the purpose of the intervention	Sub-construct C E.g. Individual specification: Whether people understand the specific tasks and responsibilities that the intervention requires of them	Sub-construct D E.g. Internalisation: Whether people can easily grasp the potential value of the intervention for their work	Neither A, B, C or D
I can distinguish the [intervention] from usual ways of working					

For the second part of the survey, respondents were asked to offer free-text comments for each construct on (a) how well a specific set of items represented each construct; (b) general comments about the items, for example, issues of item redundancy or item construction and (c) to make any general comments about the items developed. The ranked-order responses and free-text comments were imported into Excel for descriptive analysis. The results were then discussed by all the study team members to decide which items should be dropped, retained or amended.

#### Piloting final questionnaire

We piloted the questionnaire with people involved in the delivery of a single intervention. In October 2013, clinical staff involved (*n* = 10) were invited to complete the questionnaire. Staff were initially contacted via email following an initial email approach from one of the intervention leaders, with a follow-up reminder 10 days later. They were given a link to a web-based version of the questionnaire. Upon completion, we asked for additional feedback and comments on their experience of completing the questionnaire.

### Phase 3: Developing indicators (April 2012-Feb 2014)

Throughout this development stage, key conceptual and practical issues explaining how new practices become normalised emerged. Systematically unpacking these issues during the development stages enabled us to think about a separate set of implementation process indicators and structured statements - or ‘normalisation measures’ - that could be combined to generate a ‘normalisation score’. These measures were intended for internal validity assessment only, as appropriate generic assessment measures of concepts resembling normalization were not available at the time.

Generating a set of ‘normalisation measures’ involved a further iterative process of discussion and directed team level feedback. During the item development process (Table 1), whilst reviewing the qualitative data from cognitive interviews, the research team discussed how talk about outcome measurements tended to be focused on intervention-related outcomes rather than progress of the implementation. The core team considered existing tools, instruments and measures within the research literature and contacted experts in the field, but no examples of ‘normalisation’ status measures appropriate to our purpose could be identified at this time. Through subsequent discussions, the core team developed a set of potential ‘normalisation indicators’. These were then subject to feedback from the full research team, through a simple online survey. The survey asked the team members to rank the indicators in order of preference and provide feedback on the wording of each indicator. The survey comments were collated for descriptive analysis and used by the core team to decide on a final set of ‘normalisation indicators’ which were then included in the final questionnaire for testing.

## Results

### Item development

For each of NPT’s 16 sub-constructs we produced between three and seven items. Centrally, we broke down the original ideas so we could further explore features of NPT. We explored potential multiple understandings of the sub-constructs. We also sought, where appropriate, to revise some of the wording of the 16-item toolkit questions, so as to refine them in terms of simplicity and applicability. For example, the original toolkit item representing the coherence sub-construct of Communal Specification reads as ‘participants collectively agree about the purpose of the intervention’. This was broadened to include different elements of ‘understanding’ (e.g. expectations) and the idea of ‘purpose’ (e.g. success) (Table 5). Over this initial process we created a total of 112 items. During the following item reduction phase, through the individual preference exercise and through the collaborative deliberation process we reduced the numbers of items to between two and five items for each sub-construct. This left us with a total of 47 items, from this we produced our initial version of the instrument.

**Table 5:** Example original toolkit item broadened to include different elements of ‘understanding’

Construct	Communal specification	Different elements of understanding
*Coherence is the sense-making work that people do individually and collectively when they are faced with the problem of operationalizing some sets of practices.*	Whether people can build a collective understanding of the purpose of the intervention	(a). Staff in this organisation have a shared understanding of the purpose of this [intervention] (b). Staff in this organisation have shared expectations about the likelihood of the success of this [intervention]

### Item testing

Initial analysis of the first round of interview data (*n* = 18) revealed a range of challenges, some minor and individualistic; some more universal, and some more conceptually problematic (see Table 3 for coding frame). Some responses indicated overall good comprehension. Some responses lacked sufficient information to understand their reasoning but seemed logical. For example, a participant might, after answering a similarly phrased item, just say ‘again, strongly agree’, hence only reporting their level of agreement, whereas with prior questions they expanded their response and so demonstrated their comprehension. For analysis purposes, both of these types of answers were considered to represent low-level comprehension problems. Minor problems included hesitations about wording and general comprehension of items, for example, ‘Ok well I’m not quite sure what you mean [by] “from current ways of working”’. The inclusion of multiple items, which were similarly worded, raised some anticipated issues of item redundancy. With all these we could find good practical and workable solutions. We revised the wording, in an attempt to clarify the meaning and, decided to include an introduction to the questionnaire that highlighted, as we were testing the instrument, some items may appear quite similar and our aim in this phase of the project was to reduce redundancy.

The key universal issue centred on how best to manage the issue of the temporality of interventions. We asked participants to complete the instrument by thinking through a specific complex intervention of which they were part. For some, the intervention they were using to answer the instrument items through was only just being introduced, so items about later stages, of say, them being aware about whether or not ‘sufficient training and support are provided’, could not be answered as they had not got to that point in the delivery of the intervention. We were aware that this is a common problem, especially as in a real-life application, people may want to use the instrument at multiple time points, say, prior to or near the start of the deployment of the intervention as well as, the middle and end of the implementation period. We had to further ensure that items were not worded for retrospective assessment only, i.e. that they could be answered prospectively. Additionally, for Version 2, we added an additional ‘option B’ for participants, alongside the Likert scale where they are asked to agree or disagree with what is being asked (‘option A’), we also included the option of offering the response ‘not relevant at this stage’.

A more conceptually problematic challenge emerged relating to three inter-related issues. One issue was about specific groups of actors that were being referred to with expressions like ‘we’. So, for example, with an item like, ‘we have a similar understanding of the purpose of this intervention’, questions were raised about who, precisely, was part of the collective of ‘we’:

Em, who’s we? Who would we be? OK, well as a researcher to do that means my team, em, or it could mean the participants

So, in this example, typical of the dataset, the respondent is unsure whether the ‘we’ relates to the trial team or the people on the ground, the participants receiving or delivering the intervention.

A second central problem was tied to how some participants, in part through making decisions about the target of the items – e.g. the ‘whom do you mean’ problem – were offering an answer to an item or items through different or multiple roles. In response to the item, ‘I perform the tasks that are essential/necessary to making the intervention work’ we got the following response:

I think in terms of a researcher erm, (pause) that’s my job, yeh making it, perform the tasks that are essential, necessary to make the intervention work, yep but again I’m thinking, yeh that’s so part of my job, but (pause) that may be more you wanting to know that from a clinician perspective, erm, (pause), erm, well yeh as a developer yeh I absolutely am, but I still have that sense that actually I’m answering it by reinterpreting it, because I’m a researcher, I’m still thinking as I’m reading that (pause) that’s to do with clinical practice and the clinician being able to figure out actually which bits of the intervention do they have to deliver, which bits they don’t or which bits can they sort of adapt a little bit and tailor somewhat in order to, at least maintain some degree of effectiveness or even actually improve its effectiveness for their individual patient, that’s what I’m thinking.

So, in this example, they offer a response in terms of ‘what I think as someone who designed the intervention’ as well as ‘what I think in terms of someone on the ground delivering the intervention’.

Finally, for some, the questions were not relevant, or even incomprehensible, given their role. So, for example, if you are answering the question as someone who designed an intervention, being asked whether you ‘believe that participating in the intervention is just part of my job’ breaches a norm, in that, the intervention is something separate, something that goes on elsewhere and is done by others. As one participant explained:

I’m sort of getting a wee bit confused now because I’m not participating in the intervention, we are evaluating the intervention.

For some of those who positioned themselves as either designers or evaluators of the intervention, they, for good scientific reasons, did not see themselves as involved in the day-to-day work of the intervention and so such lines of questioning were problematic and confusing. Taken together these three issues raised very important questions.

At the end of this first phase of sampling and analysis we were faced with decisions about how best to manage these important problems. Over a series of weekly data-sessions with the core team, alongside teleconferences with the wider team, we established a potential conceptual understanding of the issue. Understanding the issue of role was central. For example, we knew that, at times, participants swapped roles, from, say, speaking as someone who developed the intervention to speaking as someone who actually delivers the intervention, in order to offer what was, for them, a more coherent answer. In this way they demonstrated a specific reading of the items, the items made sense to them in terms of people whose role was to deliver the intervention.

We further divided the responses to each item into three categories, that people were either speaking as ‘evaluator’, ‘observer’ or ‘doer’. Answering an item from the perspective of an ‘evaluator’ meant one was speaking from the position of someone who was involved in the planning, design, roll-out or evaluation of an intervention, often as a Principal Investigator or as a desk-based researcher. Answering as an ‘observer’, meant one was concerned with overseeing the management of an intervention, often speaking as a trial manager or NHS manager. In this way, responding to the item can involve some ‘collective averaging’ of a range of responses observed in order to make a summary judgement. Finally, speaking as ‘doer’ meant speaking from the position of directly delivering or receiving the interventions, so for example, a therapist, nurse or teacher. When all of the interview responses were grouped according to these categories we saw how and where problems remained, diminished or were eliminated. When speaking as an ‘evaluator’, at times, the problem of multiple roles remained and at times, many of the items were routinely positioned as unanswerable, either because the respondent did not have access to the information or did not see the question as relevant or appropriate. When speaking as an ‘observer’, these issues were a lot less relevant, although still appeared, briefly with some items, in part as respondents felt they did not have enough information and were therefore forced to speculate. However, when speaking as a ‘doer’, the problem of multiple roles was lost and the focus of the item was seen as relevant to this position.

For the second round of sampling, we decided to focus on asking people to complete the questionnaire taking on the role as either ‘doers’ or ‘observers’. As such, our sampling was focused on recruiting people who did this type of work. We felt asking people to speak in these roles managed the potential issue of incomprehensibility. It also set a specific direction for the questionnaire for the next round of cognitive interview, the focus would be on those who are working on the ground, those who are actively involved in either directly overseeing (‘observers’), delivering or receiving the intervention (‘doers’). In one case, given their role in an intervention, we asked someone to complete the instrument twice, going through initially as a ‘doer’ and then repeating this as an ‘observer’.

As noted above, we had already decided to add an additional ‘option B’ for each answer, ‘not relevant at this stage’. Alongside this, we also added two other option B responses in order to expand the options for participants – ‘not relevant to my role’ and ‘not relevant to this intervention’ – as we wanted the items, and the instrument itself, to be flexible and adaptable to the local circumstances of each implementation process. We also decided to remove the ‘we’ and replace it with the more directive category of ‘staff at this organisation’. Given the response to a few of the items, we altered elements of the wording of them. In six cases, we further split the item into two discrete components. For example, ‘Sufficient training and support are provided’ was split to focus on ‘training’ for one item – ‘Sufficient training is provided to enable staff to implement the intervention’ - and ‘support’ as another, separate item.

The second round of cognitive interviews (*n* = 9) revealed a similar range of minor and individualistic challenges, about wording and multiple items. However the conceptual challenges were less. We noted that those completing items as ‘observers’ were, at times, still confused or lacked the relevant information. However, those completing as ‘doers’ had no such challenges. This finding was also demonstrated in the person who was asked to complete once as a ‘doer’ and once as an ‘observer’. For them, responding as a ‘doer’, was a slightly smoother process. Again, we made some minor changes to wording, to manage minor issues of comprehension.

The third and final round of interviewing focused only on participants responding only as ‘doers’. In this round, we recruited participants (*n* = 3) who had already taken part in the first round, to complete the questionnaire again. This enabled us to check both the amendments to items as well as undertake a crude assessment of test-retest reliability. We found no substantive or minor problems and found the items to have high face validity. In this way, despite an initial focus on developing a questionnaire that could be completed by a range of people, including researchers involved in developing and evaluating an intervention, the final questionnaire was now targeted explicitly at implementation participants, the ‘doers’, those people on the ground either directly delivering or receiving an intervention. The version of the questionnaire that emerged from this process now had 53 items.

### Theoretical integrity

The next phase involved testing the theoretical integrity of the items. As a theoretically driven measurement approach, ensuring construct validity aligned with the content domains for the constructs of coherence, cognitive participation, collective action and reflexive monitoring, was essential. We obtained 23/30 survey responses (response rate 77%) from people with a good working knowledge of NPT. Descriptive analysis of responses obtained in the online survey was undertaken for each item. Alongside this, we collated the free text comments. In considering items for inclusion in the final questionnaire, individual items were examined for their perceived strength and linear relationship with sub-constructs. Through a process of consensus with the full study team, items that were deemed as poor performers, that displayed a poor linear alignment with sub-constructs were removed (*n* = 8). In this reduction process, a minimum of two items per sub-construct were retained.

Where some items were considered borderline or worthy of retaining, these were retained for revision (*n* = 6). Where items were revised or combined, the study team further deliberated the implications of the items and revisited the original theoretical constructs to inform and redress these items (See Table 6). One item that had been accidently removed from the online survey was reinstated. Table 6 shows the items that were retained, removed and revised for inclusion in Version 4 of the questionnaire (*n* = 43 items).

**Table 6:** Revisited items for inclusion in Version 4

Construct	Sub-Construct	Online expert survey items	Re-writes & exclusions	Revised Version 4
Coherence is the sense-making work that people do individually and collectively when they are faced with the problem of operationalizing some sets of practices.	*Differentiation*	(1) I can distinguish the [intervention] from usual ways of working (2) I can see how the [intervention] differs from usual ways of working (3) The [intervention] is easy to describe	(excluded) The [intervention] is easy to describe	(1) I can distinguish the [intervention] from usual ways of working (2) I can see how the [intervention] differs from usual ways of working
	*Whether people can easily understand that the intervention is different from current ways of working*			
	*Communal specification*	(4) Staff in this organisation have a shared understanding of the purpose of this [intervention] (5) Staff in this organisation have shared expectations about the likelihood of the success of this [intervention]		(4) Staff in this organisation have a shared understanding of the purpose of this [intervention] (5) Staff in this organisation have shared expectations about the likelihood of the success of this [intervention]
	*Whether people can build a collective understanding of the purpose of the intervention*			
	*Individual specification*			
	*Whether people understand the specific tasks and responsibilities that the intervention requires of them*	(6) I understand what tasks the [intervention] requires of me (7) I understand how the [intervention] affects the nature of my own work		(6) I understand what tasks the [intervention] requires of me (7) I understand how the [intervention] affects the nature of my own work
	*Internalization*	(8) I can see the potential value of the [intervention] for my work (9) I can see the worth of the [intervention] (10) I will benefit personally from being involved in the [intervention]	(re-write) I can see the worth of the [intervention] (excluded) I will benefit personally from being involved in the [intervention]	(8)I can see the potential value of the [intervention] for my work (9) I can see the worth of the [intervention] for me
	*Whether people can easily grasp the potential value of the intervention for their work*			
Cognitive participation is the relational work that people do to build and sustain a community of practice around a new technology or complex intervention.	*Initiation*	(1) There are key people who have the skills to drive the [intervention] forward (2) There are key people who drive the [intervention] forward and get others involved (3) There are individuals who enthusiastically involve others in the [intervention] (4) There are individuals who champion the [intervention]		(1) There are key people who have the skills to drive the [intervention] forward (2) There are key people who drive the [intervention] forward and get others involved (3) There are individuals who enthusiastically involve others in the [intervention] (4) There are individuals who champion the [intervention]
	*Whether key individuals are working to drive the intervention forward*			
	*Legitimation*	(5) I believe that participating in the [intervention] is a legitimate part of my role (6) The work of the [intervention] is consistent with my professional responsibilities (7) I can make an important contribution to the success of the [intervention]		(5) I believe that participating in the [intervention] is a legitimate part of my role (6) The work of the [intervention] is consistent with my professional responsibilities (7) I can make an important contribution to the success of the [intervention]
	*Whether people feel that they are the right people to be involved*			
	*Enrolment*	(8) I am able to contribute to delivering the [intervention] (9) I am willing to contribute to delivering the [intervention] (10) I can work with colleagues to deliver this [intervention]	(re-write) I am able to contribute to delivering the [intervention] (re-write) I am willing to contribute to delivering the [intervention] (re-write) I can work with colleagues to deliver this [intervention]	(8) Staff are open to new ways of working together to use the [intervention] (9) I’m open to working with colleagues in new ways to use the [intervention]
	*Whether people can organise themselves to contribute to the intervention*			
	*Activation*	(11) I feel motivated to continue to support the intervention (12) I will continue to support the [intervention] (13) Staff are willing to address any problems that arise in order to keep the [intervention] going	(excluded) I feel motivated to continue to support the intervention	(12) I will continue to support the [intervention] (13) Staff are willing to address any problems that arise in order to keep the [intervention] going
	*Whether people collectively work to support and sustain the intervention*			
Collective action is the operational work that people do to enact a set of practices, whether these represent a new technology or complex healthcare intervention.	*Interactional workability*	(1) I carry out the [intervention] tasks that are expected of me (2) I perform the tasks that are necessary to making the [intervention] work (3) The [intervention] does not make it difficult for me to do my job (4) The [intervention] makes my job easier (5) I am able to make the [intervention] a normal part of my work (6) I can easily integrate the [intervention] into my existing work	(excluded) I carry out the [intervention] tasks that are expected of me (excluded) I perform the tasks that are necessary to making the [intervention] work (excluded) The [intervention] makes my job easier	(3) The [intervention] does not make it difficult for me to do my job (5) I am able to make the [intervention] a normal part of my work (6) I can easily integrate the [intervention] into my existing work
	*Whether people can work with the intervention to perform the tasks required in their role*			
	*Relational Integration*			
	*Whether people maintain trust in the intervention and trust in each other*	(7) I have confidence in others to deliver the [intervention] (8) The [intervention] disrupts working relationships (9) The [intervention] threatens trust between staff members (10) I have confidence in other people’s ability to use the [intervention]		(7) I have confidence in others to deliver the [intervention] (8) The [intervention] disrupts working relationships (9) The [intervention] threatens trust between staff members (10) I have confidence in other people’s ability to use the [intervention]
	*Skill set workability*	(11) Work is assigned to those with skills appropriate to the [intervention] (12) Sufficient support provided to enable staff to implement the [intervention] (13) The [intervention] makes good use of all staff members expertise	(excluded) Sufficient support is provided to enable staff to implement the [intervention]	(11) Work is assigned to those with skills appropriate to the [intervention] (12) Sufficient training is provided to enable staff to use the [intervention] **NOTE – This item was accidently removed from the online survey. (13) The [intervention] makes good use of all staff members expertise
	*Whether the work required by the intervention is allocated to people with the right mix of skills and training*			
	*Contextual Integration*	(14) Sufficient resources are available to support the [intervention] (15) Management adequately support the [intervention] (16) The [intervention] fits well with strategic goals of this organisation		(14) Sufficient resources are available to support the [intervention] (15) Management adequately support the [intervention] (16) The [intervention] fits well with strategic goals of this organisation
	*Whether the intervention is adequately supported in terms of money, strategy and other resources by its host organisation*			
Reflexive monitoring is the appraisal work that people do to assess and understand the ways that a new set of practices affect them and others around them.	*Systemisation*	(1) I have access to reports about the effects of the [intervention] (2) I have access to anecdotal evidence about the effects of the [intervention] (3) I am aware of reports about the effects of the [intervention] (4) I am aware of anecdotal evidence about the effects of the [intervention]	(excluded) I am aware of anecdotal evidence about the effects of the [intervention]	(1) I have access to reports about the effects of the [intervention] (2) I have access to anecdotal evidence about the effects of the [intervention] (3) I am aware of reports about the effects of the [intervention]
	*Whether people have access to information about the impact of the intervention*			
	*Communal Appraisal*	(5) The staff agree that the [intervention] is working well (6) The staff agree that the [intervention] is worthwhile		(5) The staff agree that the [intervention] is working well (6) The staff agree that the [intervention] is worthwhile
	*Whether people, through formal information, collectively agree that the intervention is worthwhile*			
	*Individual Appraisal*	(7) Staff agree that the [intervention] is worth the effort they put into it (8) Staff feel that the effects of the [intervention] are worth the effort they put into it (9) I value the effects the [intervention] has had on my work		(7) Staff agree that the [intervention] is worth the effort they put into it (8) Staff feel that the effects of the [intervention] are worth the effort they put into it (9) I value the effects the [intervention] has had on my work
	*Whether individuals involved in the intervention think it is worthwhile*			
	*Reconfiguration*	(10) Feedback about the [intervention] can be used to improve it in the future (11) I can see where improvements can be made to the [intervention] (12) Staff members have been able to contribute to decisions about how the [intervention] is implemented	(re-write)I can see where improvements can be made to the [intervention] (re-write) Staff members have been able to contribute to decisions about how the [intervention] is implemented	(10) Feedback about the [intervention] can be used to improve it in the future (11) I can modify aspects of the [intervention] (12) I can modify how I work with the [intervention]
	*Whether individuals or groups can modify their work in response to their appraisal of the intervention*			

### Pilot testing

Five participants completed the pilot survey (response rate 50%). Completion rate of the items was 100%. As noted above, at the end of the survey, we asked for additional feedback on completing the survey. We got two responses, one noted that it was ‘straight forward’ but that it ‘didn’t display well on iPhone’. Given that such surveys are rarely a priority task and so may well be completed on mobile devices, we needed to check the compatibility of the online survey tool providers’ software across a range of devices. We got another, more theoretical response

Interesting survey. Gives me the feeling that it could raise alarm about the problems of implementing a tool and be very good at that but without ever discovering what the actual problem is.

Clearly, they do not report any problem in comprehension or any practical problems. However, the questionnaire raised, for them, a more epistemological question. They obviously see the possibility of the items to ‘raise the alarm’ that a problem exists, but feel that it fails to describe or capture, in any detail, the ‘actual problem’. In this way, the phenomena - the problem - and the detailed insiders’ knowledge of the phenomena would escape without accompanying qualitative work.

### Indicators of normalization

The research team specifically discussed the need to develop a set of indicators that aimed to capture and reflect a range of important factors including:

- intervention context (e.g. global versus site-specific);
- temporality of progression (e.g. current views, expectations about future normalisation);
- temporality of use (e.g. impact of frequency of use, impact of ease of use irrespective of time using).

The team also acknowledged that attempts to identify any one key term or phrase to represent the concept of normalisation – for example terms like ‘normal’, ‘routine’ or ‘conventional’ - would likely to only partially represent components of normalisation.

Over time, through discussion, the core team developed a set of 4 normalisation indicators. These where then sent out for review by the wider team. Through this process, we found that team members’ preference for indicators varied between concepts of normalisation (lowest average rating was for ‘routine’ and highest average rating for ‘conventional’) (Table 7) and feedback about the wording of each indicator included considering the applicability of each in terms of the timing and stages of an intervention, and the need for as few items as possible for ease whilst maintaining ‘an accurate picture of normalisation’. This resulted in the inclusion of three global normalisation items (Table 8). We also felt that this set of items could be added to by sites themselves, to include more intervention and site-specific indicators aimed at overall assessment of the intervention.

**Table 7:** Rank order preference for ‘normalisation concepts’ (scale 0–10)

‘Normalisation concept’	Rating average
Routine	2.67
Normal	2.89
Taken for granted	4.33
Accepted	5.00
Integrated into	5.67
Standard	5.78
Usual	6.11
Habitual	6.78
Typical	7.00
Conventional	8.78

**Table 8:** Global Normalisation Items

Global Normalisation items	Response options
1. When you use [intervention], how familiar does it feel?	Still feels very new (0) to Feels completely familiar (10)
2. Do you feel [intervention] is currently a normal part of your work?	Not at all (0) to Completely (10)
3. Do you feel [intervention] will become a normal part of your work?	Not at all (0) to Completely (10)

## Discussion

In this paper, we have described the development of an instrument to measure factors likely to affect normalization from the perspective of implementation participants based on Normalization Process Theory. We have revealed a deeper understanding of implementation process and practice, by highlighting the complexity and multiplicity of understandings that different stakeholders are likely to hold regarding the work of implementation and their own roles within it. The process of moving through cycles of theoretical translation, item generation, cognitive testing, and theoretical (re)validation was essential for maintaining a balance between the theoretical integrity of the NPT concepts we wished to measure and the ease with which intended respondents could answer the questions. Care had to be taken where cognitive interview data suggested a need for re-wording of items for respondents to make sense of them, yet subtle changes in wording could alter the fit between the item itself and the construct it was intended to measure.

Our approach to measure development and the results of this process add support to the call from Glasgow and Riley [18] for ‘pragmatic measures’ that balance psychometric considerations against the requirements of implementation in real life contexts. Our starting point for extending our theoretical conceptualisations into measurement items was an iterative process of usability testing from diverse stakeholders, to ensure development of a set of measures that were clearly focused on participants’ experiences – those people on the ground either directly delivering or receiving an intervention - and emphasised both content validity, and face validity, as evidenced through demonstration of respondent usability. The questionnaire development methods we used align closely with those recommended and used successfully by others, including cognitive testing; expert review of theoretically-based items and pilot testing [8, 52–54]. Although, our approach was based less on synthesis of existing literature and items, and more on end user response. Additionally, we systematically tested the readability and comprehensibility of items using appraisal systems (QAS-99 and QUAID). In particular, the use of consensus methods *throughout* all stages of the development process - translating the theoretical constructs, coding and interpreting cognitive interview responses, judging test items against construct definitions, and agreeing appropriate ‘normalisation’ indicator items for validity checking – is a real strength of this study. Further assessment will demonstrate adequacy of the NoMAD instrument to meet Glasgow and Riley’s criteria of being important to stakeholders, low burden to respondents, and actionable through strategies to address any problems identified by the measure [45]. They argue that ‘pragmatic measures’ should also meet the recommended criteria of being broadly applicable to a range of settings, unlikely to cause harm to those responding, related to theory, and psychometrically strong [18].

A key challenge for this study was that we wanted our instrument to reflect intellectual input from the range of stakeholders involved in the work of implementation – researchers, supervisors, implementers – but it became clear that the test items were being interpreted in very different ways by those in different roles in relation to the object of implementation. Indeed, those with multiple ‘hats’ in relation to an implementation project, moved between reflexive positions both in answering individual items, and across items as they worked through the instrument in cognitive interviews.

We would argue that the problems reflected on here are illustrative of the nature of collaborative work involved in implementation. Our challenges then were to (a) clarify the intended respondents for our instrument (those taking part in delivering the intervention – the ‘doers’), and (b) ensure that the instrument response options accommodated genuine reasons for inability to make ratings against some of the items. The inclusion of ‘option B’ responses for indicating that items weren’t relevant to their role, the intervention, or the stage of implementation, were thus essential for maintaining answerability of the questionnaire items and for ensuring that the quality data obtained using the rating scales was not compromised by forced completion.

The identification of a small number of appropriate ‘normalisation’ items to include in the NoMAD instrument for validity checking against the sub-construct measures, was especially challenging. At the time of commencing this study, we were unable to identify existing measures to inform our development or selection of existing items for inclusion in the testing phase of our study. Consensus about how to define ‘implementation outcomes’ and related concepts remains lacking, and measurement of these concepts is further limited. Proctor et al. [1] differentiate between ‘service system and clinical outcomes’ and ‘implementation outcomes’, and they define the latter as:

the effects of deliberate and purposive actions to implement new treatments, practices and services’ (p65)

As such, these are important as they indicate the success of an implementation effort, and can be key intermediate outcomes that can affect success in relation to clinical and service related outcomes expected in relation to a change in practice. Proctor et al. [1] reviewed the implementation literature to develop a taxonomy of eight conceptually distinct implementation ‘outcomes’: acceptability, adoption, appropriateness, feasibility, fidelity, implementation cost, penetration and sustainability. Since completing the development of NoMAD, work on implementation outcomes measurement has also advanced, highlighting the development of new measures of concepts such as penetration and sustainability [15]. Elements of all of these concepts featured in the workshops we undertook as a team to develop our own measures, but we also explored problems of normalisation measurement related to temporality. We recognised that, depending on the timing of assessment across an implementation trajectory, the questions of interest to evaluators may be around what is expected (at the beginning), what is happening now, or whether participants can anticipate an intervention becoming part of routine practice in the future.

We aimed to develop an instrument that would be primarily intended for healthcare implementation, but which could work across different contexts. Our work with those implementing health related interventions outside healthcare settings, such as education for example, revealed that commonly used language in one setting may have an entirely different meaning in another, and affect how respondents approach the items in the instrument. Careful analysis of response data sensitised us to these issues and allowed us to re-word items appropriately on most occasions, but we must acknowledge that the instrument is likely to fit more seamlessly into healthcare implementation assessment. Recent work on the impact of context on the assessment of implementation process and outcomes supports these findings [55].

The results of this study both support and extend NPT. Previously, NPT has been used as a framework for understanding implementation processes, primarily in qualitative studies [20], and these have demonstrated strong support for the descriptive power of NPT as a theory of implementation. We have previously used consensus methods to develop the 16 item toolkit [43] as a tool for ‘thinking through’ implementation projects, rather than as validated assessment measures as was the objective of this study. We also undertook a smaller scale project (TARS) [40] to develop NPT based measures of e-health implementation processes, which highlighted important issues concerning the framing of items and response scales; and the challenges of multiple stakeholder assessments. These studies in combination informed the more in-depth questionnaire development methods described here. The NoMAD questionnaire developed in this study will allow us to more formally test the stability of the NPT constructs, and the relationships amongst them, and will facilitate comparisons of implementation processes across intervention sites and over time. Some of these questions have been preliminarily addressed in the validation phase of the NoMAD study [44, 45]. NoMAD represents a measure that can be used to understand implementation participants’ experiences - those people on the ground either directly delivering or receiving an intervention. We feel that NoMAD can be used by implementation evaluators and researchers to reflect on elements of the impact of implementation activity on implementation participants. We envisage NoMAD being used alongside instruments that measure other dimensions of implementation outcomes, process and impact, such as implementation fidelity, adoption, and readiness.

This study advances implementation science, by further developing instruments for theory based measurement of implementation processes. To date, the development of reliable and practical instruments for measuring implementation processes and outcomes has been limited and instruments informed by theory are especially lacking [14–17, 56]. Studies reporting the development of instruments are generally not well-indexed in electronic databases. Reviewing instrumentation issues in the field of Implementation Science, Martinez et al. [14] encouraged greater effort towards addressing a number of challenges, including the use of frameworks, theories and models; determination of psychometric properties of instruments being developed; careful development of ‘home grown’ instruments; appropriate choice of evaluation methods and approaches; keeping instruments practical to use; and the development of decision-making tools to guide instrument choice.

## Conclusions

This study represents progress in advancing knowledge and capacity to measure implementation factors, by both providing a theory-based questionnaire that can be used to assess factors likely to affect normalization from the perspective of implementation participants - those people on the ground either directly delivering or receiving an intervention - and by sharing the process of developing the questionnaire so that others may more effectively develop instruments to meet needs that are not met by existing instruments within the field. What is not yet known is its potential for use in predicting the likely outcome of an attempt to implement an intervention, and whether NPT based instruments can be used prospectively to enhance implementation. To this end, further critical investment in the development of instruments such as the NoMAD questionnaire, and in further testing both longitudinally and in larger samples of individuals involved in implementation activities is warranted.

## Abbreviations

NoMAD: Normalization Measure Development
NPT: Normalization Process Theory
QAS-99: Question Appraisal System
QUAID: Question Understanding Aid
TARS: Technology Adoption Readiness Scale
